# The Structure of PrP^Sc^ Prions

**DOI:** 10.3390/pathogens7010020

**Published:** 2018-02-07

**Authors:** Holger Wille, Jesús R. Requena

**Affiliations:** 1 Centre for Prions and Protein Folding Diseases & Department of Biochemistry, University of Alberta, Edmonton, AB T6G 2M8, Canada; wille@ualberta.ca; 2 CIMUS Biomedical Research Institute & Department of Medical Sciences, University of Santiago de Compostela-IDIS, 15782 Santiago de Compostela, Spain

**Keywords:** PrP^Sc^, prion structure, β-solenoid, cryo-electron microscopy, prion propagation, amyloid

## Abstract

PrP^Sc^ (scrapie isoform of the prion protein) prions are the infectious agent behind diseases such as Creutzfeldt–Jakob disease in humans, bovine spongiform encephalopathy in cattle, chronic wasting disease in cervids (deer, elk, moose, and reindeer), as well as goat and sheep scrapie. PrP^Sc^ is an alternatively folded variant of the cellular prion protein, PrP^C^, which is a regular, GPI-anchored protein that is present on the cell surface of neurons and other cell types. While the structure of PrP^C^ is well studied, the structure of PrP^Sc^ resisted high-resolution determination due to its general insolubility and propensity to aggregate. Cryo-electron microscopy, X-ray fiber diffraction, and a variety of other approaches defined the structure of PrP^Sc^ as a four-rung β-solenoid. A high-resolution structure of PrP^Sc^ still remains to be solved, but the four-rung β-solenoid architecture provides a molecular framework for the autocatalytic propagation mechanism that gives rise to the alternative conformation of PrP^Sc^. Here, we summarize the current knowledge regarding the structure of PrP^Sc^ and speculate about the molecular conversion mechanisms that leads from PrP^C^ to PrP^Sc^.

## 1. Introduction

PrP^Sc^ was the first prion—i.e., infectious protein—to be discovered, and continues to be the quintessential prion, not only because of its historical preeminence, but also because of its association with a unique class of fatal diseases. PrP^Sc^ is the only prion known to date to have caused local epidemic and epizootic outbursts. Some of these have captured the attention of the public and even caused shockwaves of panic [1].

Ovine and caprine scrapie—fatal neurodegenerative ailments endemic in Europe—have been known for centuries, but it was not until the 1930s that its infectious nature was discovered [2]. Later, in the 1980s, PrP^Sc^ was identified as the infectious agent causing transmission of scrapie, and as the first prion ever, and was used to define the term “prion” [3,4]. Despite efforts to eradicate it, scrapie continues to be enzootic in Europe. However, it is not transmissible to humans, a phenomenon known as transmission barrier. The practice of industrial cannibalism resulted in PrP^Sc^ prions being recycled into cattle feed and causing bovine spongiform encephalopathy (“mad cow disease”) [1,4,5]. The ensuing bovine spongiform encephalopathy (BSE) epizootic affected hundreds of thousands of animals throughout Europe in the 1980s to 2000s. In turn, BSE PrP^Sc^ prions transmitted to humans, causing transmissible variant Creutzfeldt–Jakob disease (vCJD). Fortunately, the barrier governing transmission of BSE PrP^Sc^ to humans is quite high, which limited the number of vCJD cases to about 200, whereas millions of individuals are likely to have been exposed to BSE PrP^Sc^ by the oral route [1]. While cases of vCJD have subsided, retrospective histopathological analyses of tonsil and appendix samples suggest that thousands of individuals harbor PrP^Sc^ in their bodies, although a very long incubation time has prevented the appearance of clinical disease so far [6]. Very long incubation times have also been observed for Kuru. Kuru was an epidemic caused by human PrP^Sc^ transmitted orally through ritual cannibalism among the Fore people from Papua New Guinea beginning in the 1950s [7]. In this instance, an initial case of sporadic Creutzfeldt–Jakob disease (CJD) is suspected to have triggered the localized epidemic. CJD PrP^Sc^ is known to have been transmitted from humans to humans iatrogenically, through the reutilization of improperly decontaminated neurosurgical instruments, dura mater grafts, or treatment with cadaveric growth hormone containing traces of PrP^Sc^ [4,5]. Also, at least three cases of transmission of vCJD PrP^Sc^ through blood transfusion have been documented [8].

Another example of widespread infectious transmission of PrP^Sc^ prions is chronic wasting disease (CWD), which affects various cervid species and which is very contagious resulting in efficient horizontal transmission. CWD was first detected in the state of Colorado (USA) and has since spread through very extensive areas of North America [9], where it appears to be becoming enzootic. More recently, six cases have surfaced in Norway [10]. It is not currently known whether these are related to North American CWD or arose spontaneously in Norwegian moose, reindeer, and red deer populations. 

All in all, while substantial experimental evidence is accruing to suggest that other misfolded proteins such as Aβ, tau or α-synuclein might be prions or at least feature prion-like behavior of the affected proteins [11,12,13], PrP^Sc^ prions stand out as truly infectious, at times highly contagious, and disease-causing pathogens that command close attention.

Yet, how can a protein become infectious? Classic infectious agents reproduce because they contain nucleic acids, biomolecules that can be copied and therefore amplified. More specifically, what is copied is the primary structure of these nucleic acids, whether DNA or RNA. In contrast, propagation of prions, and more specifically, of PrP^Sc^ prions, involves reproduction not of their primary, but of their secondary, tertiary, and quaternary structures, i.e., their conformation [4]. PrP^Sc^ coerces PrP^C^, a glycosylphosphatidylinositol-anchored (GPI-anchored) membrane protein with the same primary but different secondary, tertiary, and quaternary structures, to adopt the PrP^Sc^ conformation. This likely involves complete unfolding of PrP^C^, first, followed by refolding through a series of molecular events in which PrP^Sc^ acts as a physical template (vide infra). To fully understand this process at the molecular level, it is essential to first know the structure of PrP^Sc^. This review presents a comprehensive summary of what we currently know about the structure of PrP^Sc^ and how its structure might encode a possible mechanism for its conformational replication. This mechanism also provides hints to explain the strain and transmission barrier phenomena, crucial in the epidemiology and epizootiology of PrP^Sc^.

## 2. The Architecture of PrP^Sc^ Prions

**Figure 1:**
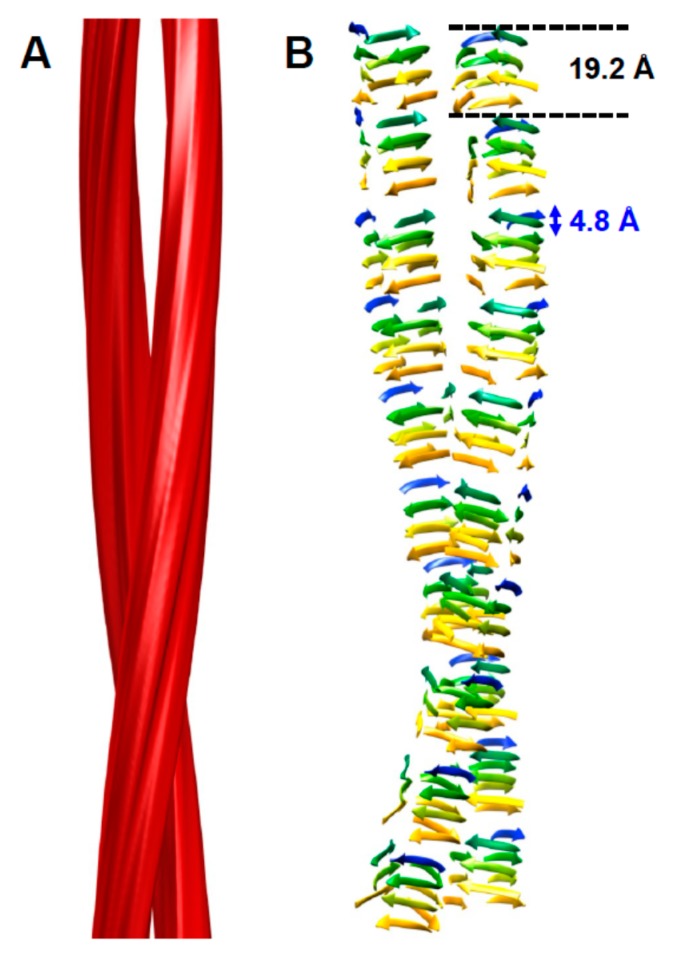
Four-rung β-solenoid architecture of PrP^Sc^. (**A**) Three-dimensional reconstruction of a PrP^Sc^ amyloid fibril with two protofilaments. (**B**) Cartoon representation of a four-rung β-solenoid architecture drawn to approximate the 3D reconstruction in (**A**). The 4.8 Å spacing of individual β-strands running perpendicular to the fibril axis is indicated, as is the 19.2 Å height of an individual PrP^Sc^ molecule. Figure adapted from Vázquez-Fernández et al. PLoS Pathog. 2016, 12, e1005835 [14].

**Figure 2:**
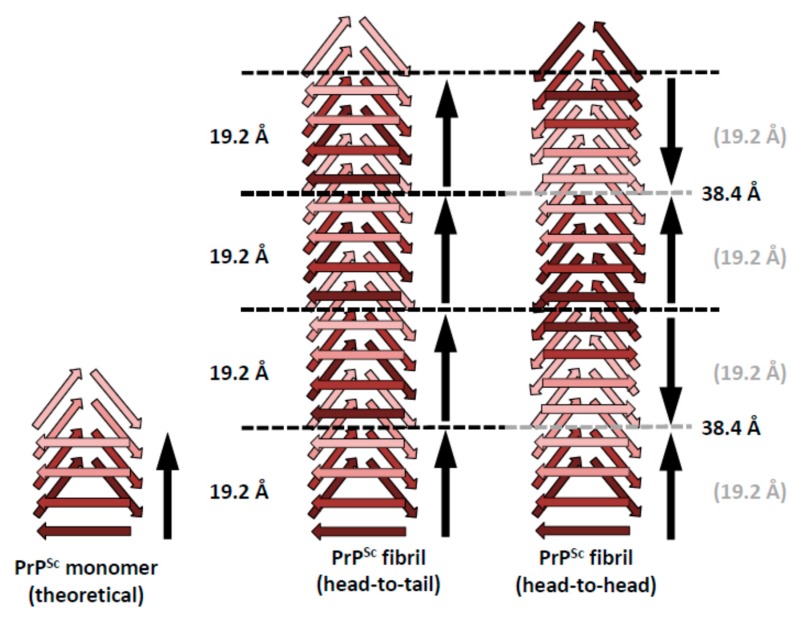
Schematic representations of possible head-to-tail and head-to-head (tail-to-tail) architectures for PrP^Sc^ amyloid fibrils. The different architectures would lead to a polar fibril in the case of head-to-tail stacking, while a head-to-head (tail-to-tail) architecture would give rise to a non-polar fibril. The ~40 Å signal that was obtained with the single particle image processing approach [14] would favor a head-to-head arrangement, since there is no straightforward mechanism by which a head-to-tail arrangement would produce such a spacing.

The structure of PrP^Sc^ is based on a four-rung β-solenoid architecture (Figure 1), as was revealed recently by cryo-electron microscopy and three-dimensional (3D) reconstructions [14]. By analyzing 3D reconstructions from individual PrP^Sc^ amyloid fibrils, and by taking the molecular density of PrP^Sc^ into consideration [15], it was possible to determine the average molecular height of each PrP^Sc^ molecule along the fibril axis as ~17.7 Å [14]. Individual measurements ranged from 16.1 to 19.25 Å, while a four-rung β-solenoid architecture would be expected to have a height of 19.2 Å (=4 × 4.8 Å). A single particle approach, which was used to average data from a much larger number of PrP^Sc^ amyloid fibril segments, produced molecular height peaks around 20 and 40 Å [14]. The former was in good agreement with the results obtained from individual amyloid fibrils, while the latter measurement suggested a larger assembly unit along the fibril axis, encompassing two monomers in a potential head-to-head/tail-to-tail configuration (Figure 2). While the cryo-electron microscopy data helped to decipher the overall architecture of PrP^Sc^ as a four-rung β-solenoid, the resolution was not sufficient to resolve the structure in atomic details. 

A β-solenoidal core was originally proposed by one of us as the key architectural element of PrP^Sc^, based on electron crystallography studies of 2D crystals from the N-terminally truncated PrP^Sc^ (PrP27-30) [16,17]. By comparing 2D projection maps from PrP27-30 2D crystals with those of an even smaller “mini-prion”, PrP^Sc^106 [18], the structure of PrP^Sc^ was constrained to contain a β-helix or β-solenoid structure at its core. At the time, it was assumed that PrP^Sc^ would contain residual α-helix structure at the C-terminus, an interpretation which is not supported by more recent experimental evidence [14,19,20].

X-ray fiber diffraction from brain-derived PrP27-30 and PrP^Sc^ amyloid fibrils gave a series of meridional diffraction signals at 9.6, 6.4, and 4.8 Å, which correspond to the second, third, and fourth order diffraction, respectively, of a 19.2 Å β-sheet structure [21]. The equatorial diffraction signatures were equally informative, in that a prominent ~10 Å signal, which is characteristic for generic stacked β-sheet amyloid structures, including those present in recombinant PrP amyloid fibrils [21,22], was absent. This absence is a strong indicator that the underlying architecture is that of a β-solenoid, as demonstrated by comparison with diffraction results obtained from the HET-s amyloid [23], which has been shown by solid state NMR to contain a two-rung β-solenoid structure [24]. All together, the X-ray fiber diffraction results provided clear evidence that the structure of PrP^Sc^ contains a four-rung β-solenoid fold at its core [21]. Subsequent analyses indicated that shorter fragments of PrP could either adopt a generic stacked β-sheet structure or shorter β-solenoids [25]. The shortest form of PrP that could support the formation of transmissible prions, PrP^Sc^106, was also found to contain a four-rung β-solenoid fold [18,25]. A number of other studies have obtained high-resolution structures from short, PrP-based peptides, often adopting “steric zippers” or related structures, but those short peptides have no biological relevance and lack the structural complexities that characterize PrP^Sc^.

Moreover, the β-solenoidal structure agrees with a number of structural restraints gathered through the years with a variety of biophysical and biochemical methods. Fourier-transform infrared (FTIR) spectroscopy, and circular dichroism spectroscopy (CD), had demonstrated a high β-sheet content of PrP^Sc^ and its N-terminally truncated variant, PrP27-30 [19,26,27,28,29]. More specifically, FTIR-based estimates of β-sheet in PrP27-30 range from 43–61% [26,27,28]. For some time, the FTIR data were interpreted to imply that PrP^Sc^ and PrP27-30 contained a substantial fraction of α-helical structure. However, Smirnovas et al. have shown that the ~1660 cm^–1^ band in the FTIR spectra of PrP^Sc^ and PrP27-30, which had been attributed to α-helices based on calibration using globular proteins, is also present in the spectrum of amyloid fibrils formed by recombinant prion protein (recPrP) known to exhibit a parallel in-register β-structure and to be completely devoid of α-helices [19]. Furthermore, the ~1660 cm^−1^ FTIR band overlaps with bands in the same region arising from turns and coils. Therefore, it can be safely concluded that the FTIR-based data do not support the presence of α-helices in PrP^Sc^ [20]. To sum up, all these studies suggest that PrP27-30 consists of about 50% β-strands and 50% random coil loops; this fits very well a four-rung β-solenoid with short β-strands connected by loops. It should be noted that the HET-s(218–289) prion, whose structure conforms to a two-rung β-solenoid and is probably quite similar to PrP^Sc^, contains ~53% of β-strands connected by ~47% loops and turns [24].

The extremely compact nature of the β-solenoid core, spanning virtually from the N- to the C-terminus of PrP27-30 (~90 to 230) is also in good agreement with the known resistance of PrP27-30 to protease digestion. Classically, when PrP^Sc^ of most strains is subjected to proteinase K (PK) treatment, its N-terminal residues, up to position 86/98 depending on the strain, is readily digested. This portion is therefore believed to retain the completely unfolded secondary structure that it exhibits in PrP^C^ [4]. It is noteworthy that this region is totally dispensable for infectivity, and therefore it can be considered as not being part of the “prionic domain” of PrP^Sc^. Nevertheless, insertions and deletions in the N-terminal domain are known to cause familial prion diseases [30]. The C-terminal domain, which corresponds to the β-solenoid part of PrP^Sc^, forms the core of the PrP^Sc^ amyloid fibril from which the N-terminal 23-86/98 “tail” projects into the medium. 

On the other hand, the existence of connecting loops, would explain the presence of small quantities of smaller PK-resistant fragments besides PrP27-30 [31,32,33,34]. Any β-solenoidal protein subjected to treatment with a relatively non-specific protease such as PK would be expected to undergo partial cleavage at the more flexible, less compact coils connecting the β-strands, while these would be relatively spared [35,36]. The extraordinary resilience of PrP^Sc^ to PK observed in early studies [4,37] suggests that the PrP27-30 β-solenoid is extremely compact, i.e., its connecting loops and turns are likely very tightly packed against the β-strands that make up the solenoid core. This would result in the scarcity of PK cleavages at these sites as compared to the fast, extensive, and complete digestion of the unfolded N-terminal stretch. However, secondary cleavages within the β-solenoid core have indeed been progressively unveiled in many PrP^Sc^ samples, particularly as a wider variety of antibodies has been used to probe the PK digests [31,32,33,34]. Furthermore, a number of PrP^Sc^ strains with increased susceptibility to PK have been identified, including Variably Protease Sensitive Prionopaty (VPSP) PrP^Sc^ [38,39], or PrP^Sc^ from spontaneously ill transgenic bank voles overexpressing PrP with the 109I polymorphism [40,41]. Also, shorter PK-resistant PrP^Sc^ fragments have been identified as being characteristic of many “atypical” PrP^Sc^ strains [39,42]. It is noteworthy that many of these strains of PrP^Sc^ are characteristically cleaved by PK at a position around ~150–153 [39]. The same cleavage has been identified as one of the most prominent secondary internal cleavages within PrP^Sc^ of “classic” strains [31,32,33,34,36]. This strongly suggests that the ~150–153 region corresponds to an important loop, perhaps connecting different rungs of the solenoid, a hinge of sorts. 

Minor differences in threading (vide infra) and/or lateral packing of loops would obviously result in very significant changes in susceptibility of different strains of PrP^Sc^ to proteolysis. 

## 3. Other Models of PrP^Sc^

Before confluent X-ray fiber diffraction and cryo-EM studies defined the β-solenoid as the basic architectural element of PrP^Sc^, a number of structural models were put forward. We have extensively reviewed them and their shortcomings [20]. Here, we will just refer to a recent one, which has received more attention due to its similarity to recently solved amyloid structures: the parallel in-register intermolecular beta-sheet model (PIRIBS) [43]. In this model, each molecule of PrP stacks on top of the preceding molecule perfectly in register. Hence, a single molecule of PrP contributes just 4.8 Å in height to the rise of a PrP amyloid fibril. Given the size of PrP, a single molecule would have to cover both “protofilaments” in the observed amyloid fibrils. As a consequence, the “protofilaments” that can be observed in electron micrographs of PrP^Sc^ amyloid [14,21,44] would not exist as separate entities and represent merely imaging artifacts. Interestingly, X-ray fiber diffraction suggested a PIRIBS-like conformation for recombinant PrP amyloid that was found to be non-infectious [21,22].

**Figure 3:**
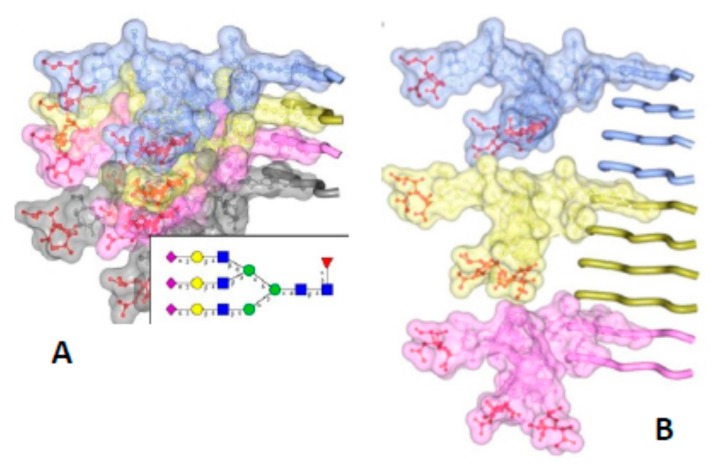
The bulky, N-linked glycans impose spatial constraints on folding patterns of PrP^Sc^ and rule out a flat, in-register stacking. Cross β-sheet structures carrying tri-antennary N-glycans (shown in inset) on each successive β-strand (**A**) or every fourth β-strand (**B**). Polypeptide chains are represented in tube form, whereas the N-glycans are shown as a combination of ball-and-stick and volume representations. Each PrP molecule with corresponding N-glycan is rendered a different color. Sialic acid residues are colored in red; N-glycan electrostatic surfaces are semi-transparent. To model the dimension of cross-β structures, the Authors of the original figure (see below) adapted the parallel beta-sheet model from PDB database entry 2RNM, an NMR structure for HET-s(218–289) prion in its amyloid form [24]. The structure of a tri-antennary N-linked glycan was taken from PDB entry 3QUM, a crystal structure of human prostate specific antigen (PSA) [50]. Adapted from Baskakov, I.V. and Katorcha, E. Frontiers in Neuroscience 2016, 10, 358 [45].

The PIRIBS model is incompatible with the height measurements from both the X-ray fiber diffraction experiments [21], and the cryo EM observations [14]. Both had independently indicated the height of a molecule of PrP^Sc^ to be 19.2 Å (see above). Furthermore, the dense stacking of the PIRIBS model cannot accommodate the bulk of the glycosylation side chains [45], which, due to their bulk, take up more space than is available in a tight packing as the PIRIBS model requires (Figure 3).

## 4. Implications of the Structure of PrP^Sc^ for Its Propagation

**Figure 4:**
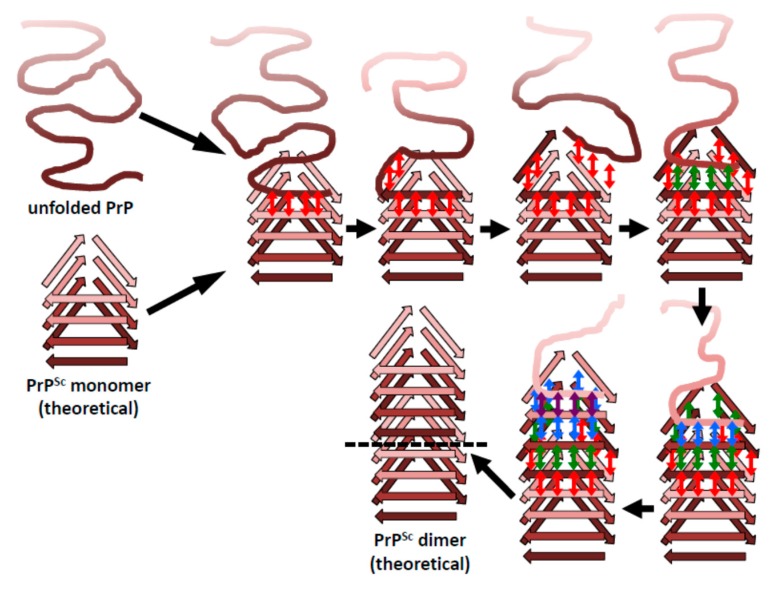
The β-solenoid architecture of PrP^Sc^ suggests a mechanism to template incoming molecules of unfolded PrP onto the existing β-solenoid fold to generate a copy of itself. For simplicity, this mechanistic model is based on a head-to-tail arrangement. An incoming molecule of unfolded PrP would interact with an uncapped β-solenoid surface and adopt a β-strand conformation by forming backbone hydrogen-bonds (red arrows). Once the first rung of a nascent β-solenoid configuration has been formed, it would self-template successive rungs of β-solenoid structure using the same mechanism (green, blue, and purple arrows, respectively). Once the fourth and final rung has been templated, a new molecule of PrP^Sc^ is formed and the original template surface has been re-created. Any mutations facilitating unfolding of PrP^C^ would lead to increased chances of propagation events.

While only the overall architecture of PrP^Sc^ has been deciphered and important structural elements are still undefined (Figure 1), it is now possible, for the first time, to formulate a sound hypothesis about how PrP^Sc^ prions propagate [46]. A β-solenoid has inherent templating capabilities: its upper- and lowermost rungs contain “unpaired” β-strands that can propagate their hydrogen-bonding pattern into any amyloidogenic peptide they encounter [47]. In fact, the edge strands of native, soluble proteins that contain a β-solenoid have evolved to be capped by loops or α-helices that block unregulated β-sheet propagation. Furthermore, when these capping structures are eliminated by means of protein-engineering techniques, the resulting “decapped” β-solenoids become unstable and undergo edge-to-edge-driven oligomerization [48]. Therefore, the upper- and lowermost β-solenoid rungs in PrP^Sc^ can template an incoming, unfolded PrP molecule, and mold it into an additional β-solenoid rung (Figure 3). Once this supplementary β-solenoid rung is formed, it offers a fresh, “sticky” surface that can continue templating the remaining, unfolded portion of the incoming PrP molecule, until a second rung is generated. This process can be repeated two more times until the entire length of the incoming PrP polypeptide chain has been molded into four newly formed rungs, thus completing a new four-rung β-solenoid structure (Figure 4). The newly formed upper- or lower-most rungs can now serve as a fresh templating surface for a new incoming unfolded PrP molecule, in a process that can proceed ad infinitum. As already mentioned above, the presence of bulky carbohydrate chains in the incoming unfolded PrP molecule must certainly impose constraints to the templating process; in turn, the GPI moiety present both in the incoming PrP molecule and in the PrP^Sc^ template likely anchoring them to the cell membrane and/or endocytic vesicle milieu, probably impose constraints relative to the cellular location(s) in which conversion takes place. Templating must necessarily be based on either a head-to-tail or a head-to-head/tail-to-tail orientation. In the former case, templating of β-sheets would involve heterotypic contacts between different parts of the molecule, while the latter would involve homotypic contacts. The structural arguments and data favoring one or the other of these two possibilities will be discussed in the following section.

How are strain and transmission barrier properties encoded in the β-solenoid architecture of PrP^Sc^? Slightly different threading, resulting in slight differences in the amino acid composition of the β-strands and loops are an obvious source of variability giving rise to different variations of the main β-solenoid theme, as already noted by Langedijk and colleagues [49]. These variations, affecting the topography of the upper- and lowermost rungs, would have an obvious impact on the templating properties of a given β-solenoid variant (strain), as depicted in Figure 3. Thus, the presence in the templating surfaces of charged or bulky amino acid residues might impose restrictions to their ability to receive and template a PrP chain of a given amino acidic sequence. This is particularly evident with respect to the sequences at the N- and C-termini of the unfolded PrP substrate, the first stretches that need to adapt to these templating surfaces; but also for the rest of the sequence, as every new rung template generates a fresh templating surface with its own steric and charge constraints. Ultimately, a higher resolution structure of PrP^Sc^ should allow to fully understand these properties of PrP^Sc^ at the molecular level.

It is noteworthy that the molecular forces responsible for templating, namely, hydrogen-bonding, charge interactions, aromatic stacking, and steric constraints, are essentially the same as those operating during DNA replication. However, they lack its exquisite precision and the complex proofreading mechanisms that provide the flexibility of nucleic acid replication. The higher complexity of the PrP^Sc^ structure, as compared to that of nucleic acids, will require particular efforts to achieve a complete understanding of all the molecular aspects associated with propagation of PrP^Sc^ prions.

## 5. Head-to-Head or Head-to-Tail Stacking?

The four-rung β-solenoid architecture of PrP^Sc^ implies two possible stacking modes: a head-to-tail stacking, resulting in polar fibril assemblies, or a head-to-head stacking, which would produce non-polar fibril assemblies (Figure 2). A head-to-head stacking mode would result from a templating process in which the first rung formed is in-register with respect to the templating rung, whereas subsequently formed rungs are not. This initial in-register stacking seems a very elegant and simple option. However, it adds the complication of successive PrP^Sc^ subunits with opposite handedness/twist, which would add an unusual level of complexity to the structure of PrP^Sc^. At the moment, the experimental evidence of roughly four-nanometer axial repeats in single particle averages from PrP^Sc^ fibrils slightly favor a head-to-head stacking [14], but the evidence is not strong enough to unequivocally resolve the question. Furthermore, it should be noted that a similar “vertical pairing” signal characteristically appears in Fast Fourier Transforms from Het-s prion fibers [15], in which templating and stacking are unequivocally known to be head-to-tail [24].

In contrast, a head-to-tail stacking would entail templating between heterotypic sequences, lacking the elegance and simplicity that in-register stacking/templating would provide. On the other hand, this propagation mode would not result in the opposite handedness/twist problems, as discussed for head-to-head templating. Intrinsically, the head-to-tail stacking would result in a prominent 19.2 Å periodicity, based on the molecular height of PrP^Sc^ (see above). Any larger spacings along the fibril axis would require alternative explanations, which cannot be provided based on currently available data.

## 6. Concluding Remarks and Outlook

Elucidation of the architecture of PrP^Sc^ allows at last to understand the molecular underpinnings of the propagation of this lethal prion. It is noteworthy that the mechanisms involved are not too different from those at play during replication of DNA: hydrogen bonding and steric fitting. However, while for DNA all the templating information can be deduced from and therefore stored in its primary structure, and can therefore be seen as “digital”, for PrP^Sc^ it involves secondary and tertiary and maybe even quaternary structure levels, and therefore it can be viewed as “analog”. Hence, it is not surprising that nucleic acids and not prions have been selected as the main elements of heredity, although prebiotic templating of amyloid has been suggested as an early precursor of cellular life [51]. The basic understanding of the four-rung β-solenoid also hints at a possible explanation of the strain and transmission barrier phenomena: strains are likely to correspond to minor variations in the threading of the solenoid, while transmission barriers are likely the consequence of steric hindrances arising from differences in the sequence of the incoming and templating PrP molecules. However, to fully understand these phenomena it will be necessary to refine our current understanding of the structure of PrP^Sc^ through higher resolution data. Further studies to that end, using improved cryo-EM techniques and NMR applied to recombinant PrP^Sc^ are being currently carried out in our and other laboratories.
